# DIETARY INTERVENTIONS FOR HEALTHY AGING

**DOI:** 10.1093/geroni/igac059.363

**Published:** 2022-12-20

**Authors:** Sai Krupa Das, Leanne Redman

**Affiliations:** Tufts University, Boston, Massachusetts, United States; Pennington Biomedical Research Center, Baton Rouge, Louisiana, United States

## Abstract

Aging is associated with a host of cellular and molecular changes that cumulatively result in a progressive decline in metabolic and physical function, development of chronic disease, and increased risk of mortality. Dietary interventions targeting these age-related changes have been shown to attenuate the aging process and improve healthspan, i.e., the length of time individuals are disease- and disability-free. Calorie restriction (CR) is one such intervention that has been shown to be effective in reducing disease risk and improving multiple markers of biological aging. The biological mechanisms mediating the observed benefits of CR are not fully understood but possibly involve changes in energy metabolism, oxidative damage, insulin sensitivity, inflammation, and function of both the neuroendocrine and sympathetic nervous systems. Despite the benefits of CR, sustained adherence remains a challenge. An alternative dietary approach with potential for impact on markers of aging and disease risk is intermittent fasting (IF), which represents a broad class of meal-timing interventions that involve alternating periods of eating and extended fasting. One novel form of IF called time-restricted eating (TRE) involves eating within a 10-hour period and fasting for the rest of the day. TRE appears to improve adherence to IF and especially improve cardiometabolic health in humans. Studies in rodents and pilot studies in humans have found that TRE reduces body weight and hunger, improves insulin sensitivity, lowers blood pressure, reduces inflammation and oxidative stress, improves circadian rhythms, and, in rodents only, extends lifespan. With feasibility data demonstrating reasonably high adherence rates, TRE is a promising strategy to improve markers of healthspan.

